# Silver Quantum Dot Decorated 2D-SnO_2_ Nanoflakes for Photocatalytic Degradation of the Water Pollutant Rhodamine B

**DOI:** 10.3390/nano9111536

**Published:** 2019-10-30

**Authors:** Nadavala Siva Kumar, Mohammad Asif, T. Ranjeth Kumar Reddy, Gnanendra Shanmugam, Abdelhamid Ajbar

**Affiliations:** 1 Department of Chemical Engineering, King Saud University, P.O. Box 800, Riyadh 11421, Saudi Arabia; masif@ksu.edu.sa (M.A.); aajbar@ksu.edu.sa (A.A.); 2 Department of Physics, Presidency University, Bengaluru 560064, India; ranjithreddy155@gmail.com; 3 Department of Biotechnology, Yeungnam University, Gyeongsan, Gyeongbuk 38541, Korea

**Keywords:** silver quantum dots, 2D-SnO_2_ nanoflakes, photocatalytic activity, rhodamine B

## Abstract

Decoration of 2D semiconductor structures with heterogeneous metal quantum dots has attracted considerable attention due to advanced optical, electrical, and catalytic properties that result from the large surface-to-volume ratio associated with these structures. Herein, we report on silver quantum dot decorated 2D SnO_2_ nanoflakes for the photocatalytic abatement of water effluents, the synthesis of which was achieved through a straightforward and mild hydrothermal procedure. The photocatalysts were systematically investigated using UV–Vis, XRD, electron microscopy (SEM, HR-TEM), EDX, XPS and FTIR. The photocatalytic activity of the nanostructures was evaluated for the abatement of water pollutant rhodamine B (RhB), under light irradiation. The mild hydrothermal synthesis (100 °C) proved highly efficient for the production of large scale Ag quantum dot (QD)/SnO_2_ nanoflakes for a novel photocatalytic application. The decoration of SnO_2_ with Ag QDs significantly enhances the synergetic charge transfer, which diminishes the photo-induced electron-hole reunion. Moreover, the plasmonic effect from Ag QDs and 2D-SnO_2_ structures acts as an electron tank to collect the photo-induced electrons, generating a Schottky barrier between the SnO_2_ structures and quantum dots. Overall, this resulted in a facile and efficient degradation of RhB, with a rate double that of pristine SnO_2_.

## 1. Introduction

The preparation of heterostructured 2D-nanosized oxide materials with wide bandgaps has been an active area of research for the past three decades, as their structural shape and size influence their physical, chemical, optical, catalytic, and electronic properties [1,2,3,4]. In particular, 2D semiconductors (graphene oxide, g-C_3_N_4_, cobalt oxide, ZnO nanosheets, TiO_2_, MoS_2_, WS_2_, and SnO_2_) have been used as photocatalysts for the improved treatment of polluted water [5,6]. In recent years, among the wide bandgap semiconductors, SnO_2_ nanomaterials have risen to the fore due to favorable properties such as a very large bandgap (*E*g = 3.6 eV at 27 °C), good chemical and biological inertness, non-toxicity, high electron mobility, environmental friendliness, and low cost and ease of production [7,8]. Owing to the above-mentioned properties, SnO_2_ nanomaterials have numerous applications in gas sensors, solar cells, lithium ion batteries, and catalysts [9,10,11,12,13,14,15]. For low dimension SnO_2_ nanostructures, various morphologies such as zero dimensional nanoparticles, one dimensional nanorods, nanowires, nanotubes, two dimensional nanosheets, and 3D hierarchical self-assembled nanostructures have been reported [16,17,18]. Among those, due to their very large fraction of distinct exposed surface facets, 2D nanosheets are favorable for applications in catalysis and photocatalysis [7,8]. However, despite the good performance of SnO_2_ as a photocatalyst, its efficiency is generally low due to the facile recombination rate of photo-generated electron-hole pairs, and its failure to function within the visible region of light [19]. It has been suggested that the doping of SnO_2_ with plasmonic noble metal nanoparticles (NPs) could overcome this limitation, as the combination of wide bandgap semiconductor nanostructures and plasmonic noble metals has excellent properties such as increased light absorption and fast transfer of photo induced charge carriers; these properties stem from the surface plasmon resonance (SPR) of noble metals [20,21]. Such a combination allows the photo-generated electrons to delocalize over the excitation levels of both the dopant and the semiconductor. The above-mentioned properties may reduce the recombination of photo-induced holes and electrons, resulting in an increased availability of these carriers to degrade water pollutants [22]. In recent years, researchers have investigated SnO_2_ nanomaterials doped with different metal nanostructures. J. Pan et al. fabricated carbon quantum dot (QD) modified SnO_2_ nanotubes for photocatalysis applications achieved within the visible spectrum [14]. Dutta et al. synthesized SnO_2_ QDs decorated with SiO_2_ nanoparticles and studied the degradation of methyl blue (MB) dye under visible light [13]. M. Hu et al. synthesized Ag decorated SnO_2_ microspheres and evaluated their catalytic performance for the conversion of 4-nitrophenol to 4-aminophenol [8], and Babu et al. reported on Ag–SnO_2_ quantum dots (plasmonic photocatalyst) for the reduction of water pollutants and studied the effect of Ag composition on photo catalytic efficiency [23].

To the best of our knowledge, Ag QD decorated 2D SnO_2_ nanoflakes/sheets have not been reported thus far. As above-mentioned, due to the large surface area of distinct exposed facets, 2D nanosheets are favorable for photocatalytic applications. With these factors in mind, we synthesized Ag QD decorated 2D SnO_2_ nanoflakes, and studied their structural, optical, and photo-catalytic properties.

## 2. Materials and Methods

Tin (II) chloride dihydrate, glucose, silver nitrate, and organic dye (RhB) were purchased from Sigma-Aldrich (St. Louis, MO, USA), and hexadecylamine (HDA) was procured from TCI Chemicals (Seoul, Korea).

Ag QDs/2D-SnO_2_ nanoflakes were synthesized by the following procedure: tin chloride dihydrate (100 mg), silver nitrate (20 mg), and HDA (180 mg) were added to deionized water (20 mL) in a 20 mL vial. Then, glucose (100 mg) was added to the solution and the resulting mixture was stirred at 35 °C for 2 h. The glass vial was then wrapped with paraffin tape, and oven heated at 102 °C for 12 h. During the formation of the 2D-nanoflakes, the amine moiety of HDA binds to the surface of tin oxide in the aqueous medium. For comparison, the same synthetic procedure was used to fabricate 2D-SnO_2_ nanoflakes, with the exception of silver nitrate addition.

Morphological features and the shapes and sizes of fabricated structures were studied using scanning and transmission electron microscopes (SEM, S-4800, Hitachi, Tokyo, Japan; FE-TEM, FEI Tecnai G2 F20, respectively). Optical studies were performed using UV–Vis spectroscopy (Jobin Varian Carry 5000 spectrophotometer). The crystallinity of the prepared 2D structures was analyzed by powder x-ray diffraction (PXRD) and the results obtained were compared with standard data. Oxidation states and elemental composition were analyzed using energy dispersive x-ray spectroscopy (EDX) and x-photoelectron spectroscopy (XPS, Thermo Scientific-Al K α1 radiation source).

Photocatalytic reactions were conducted under artificial solar irradiation (A Solar simulator, ABET technologies, LS series light source, Gyeonggi-do, Korea), and the average light intensity was calculated at approximately 120,000 lux, which was measured by a digital lux meter. The photocatalytic reduction kinetics of RhB (5 ppm) were determined under an artificial solar illuminator in aqueous media. The prepared catalyst (20 mg) was dispersed in a prepared dye solution (100 mL) by stirring the mixture for 30 min in the dark, in order to maintain the adsorption/desorption stabilities of the dye and catalyst. Degradation kinetics were measured with a UV–Vis absorption spectrophotometer (Thermo Scientific Genesys10S).

## 3. Results and Discussion

**Figure 1:**
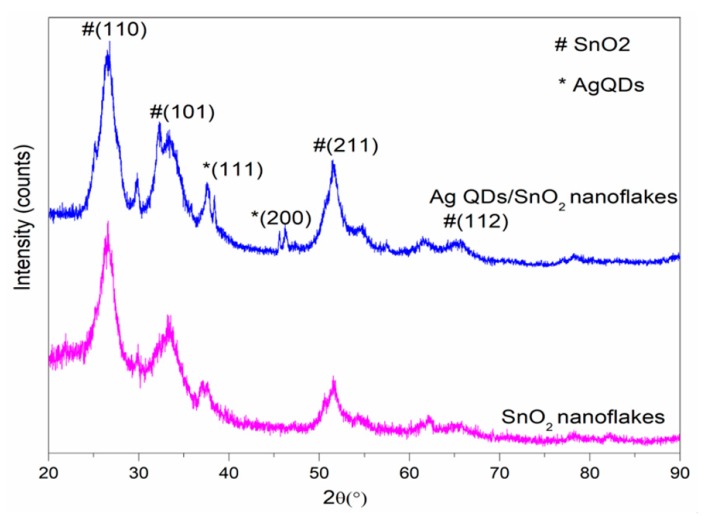
Powder x-ray diffraction (PXRD) spectra of the SnO_2_ and Ag QD/SnO_2_ nanostructures.

The crystalline nature of the Ag QD decorated 2D SnO_2_ nanoflakes and pristine 2D-SnO_2_ nanoflakes were investigated by PXRD, and the results are represented in Figure 1. The results revealed that the characteristic Bragg diffraction peaks correlated well with the tetragonal rutile phase of SnO_2_ (JCPDS NO: 41-1445); the patterns of Ag QD decorated SnO_2_ contained peaks for both SnO_2_ and Ag, with the diffraction peaks of Ag matching well with the face centered cubic facets of Ag (JCPDS-04-0783). Moreover, the low content of silver in the Ag QD decorated SnO_2_ gave rise to small, intense significant diffraction peaks associated with the silver, due to the silver atoms anchoring on SnO_2_ lattice planes. Likewise, the presence of additional peaks at the smaller angles was attributed to the attachment of Ag ions with Sn ions.

**Figure 2:**
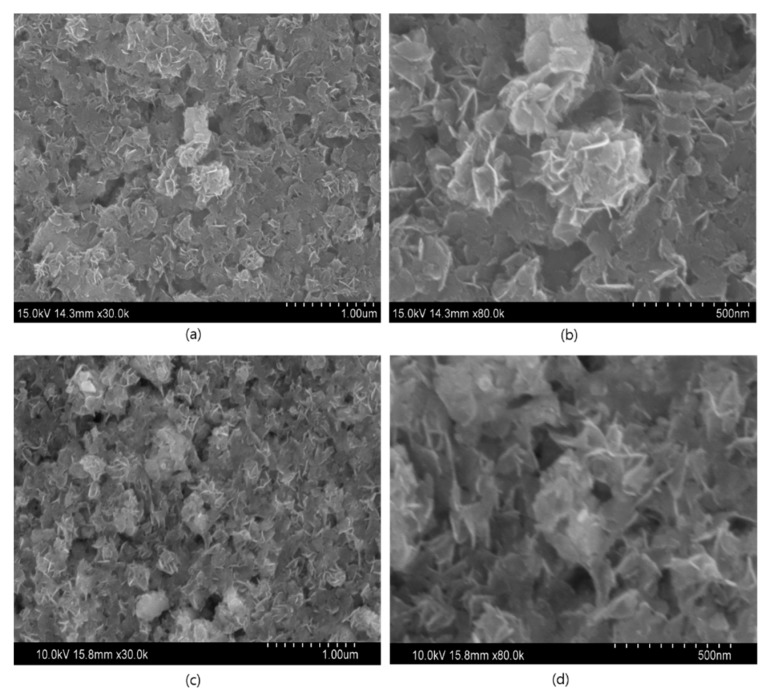
Field emission scanning electron microscopy (FESEM) images (**a**,**b**) SnO_2_ nanoflakes (**c**,**d**) Ag QDs/SnO_2_ nanostructures.

**Figure 3:**
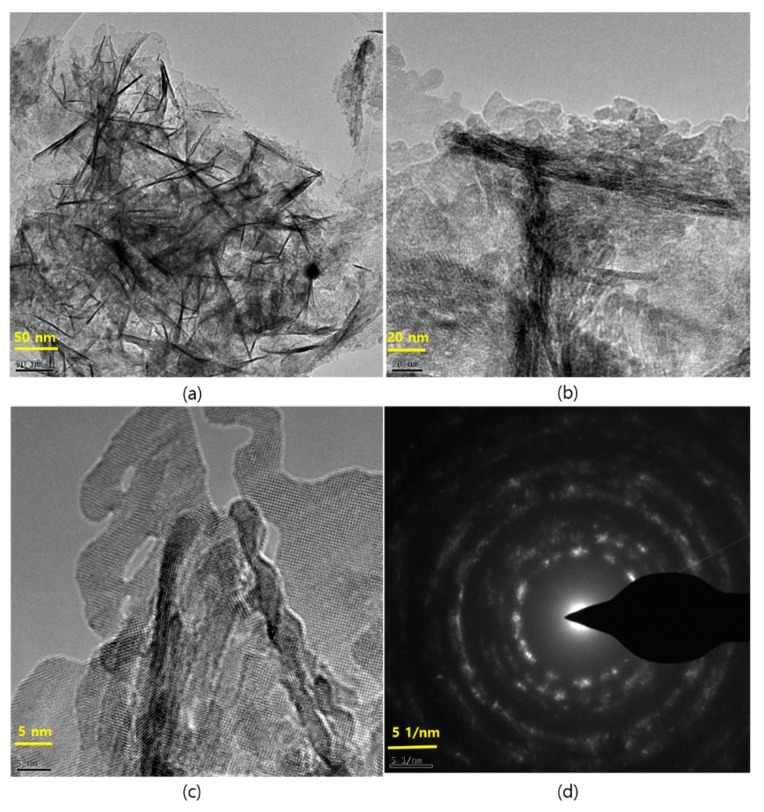
TEM images at different magnification of the Ag QDs/SnO_2_ nanostructures and their selected area electron diffraction (SAED) pattern. (**a**) Ag QDs/SnO_2_ nanostructures at 50 nm; (**b**) Ag QDs/SnO_2_ nanostructures at 20 nm; (**c**) High resolution image Ag QDs/SnO_2_ nanostructures at 5 nm; (**d**) SAED pattern of Ag QDs/SnO_2_ nanostructures.

**Figure 4:**
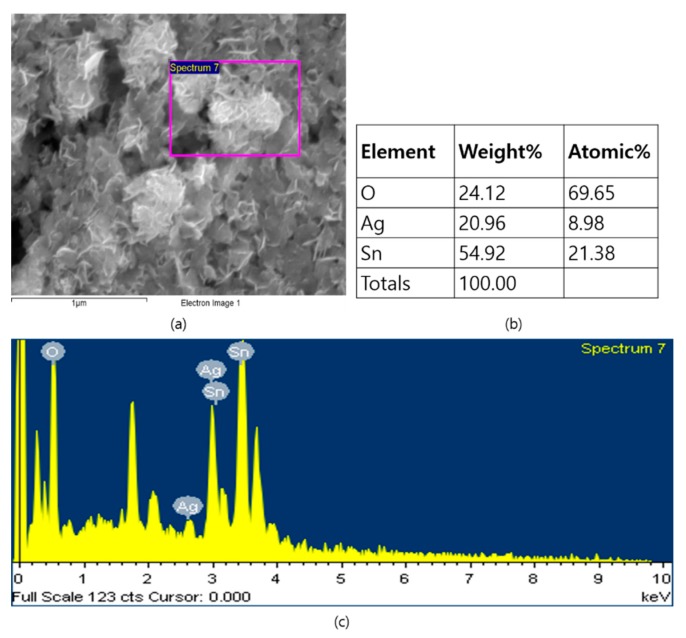
EDX spectrum of the Ag QDs/SnO_2_ nanostructures: (**a**) SEM image; (**b**) EDX data of Ag QDs/SnO_2_ nanostructures; (**c**) EDX spectrum of Ag QDs/SnO_2_ nanostructures.

**Figure 5:**
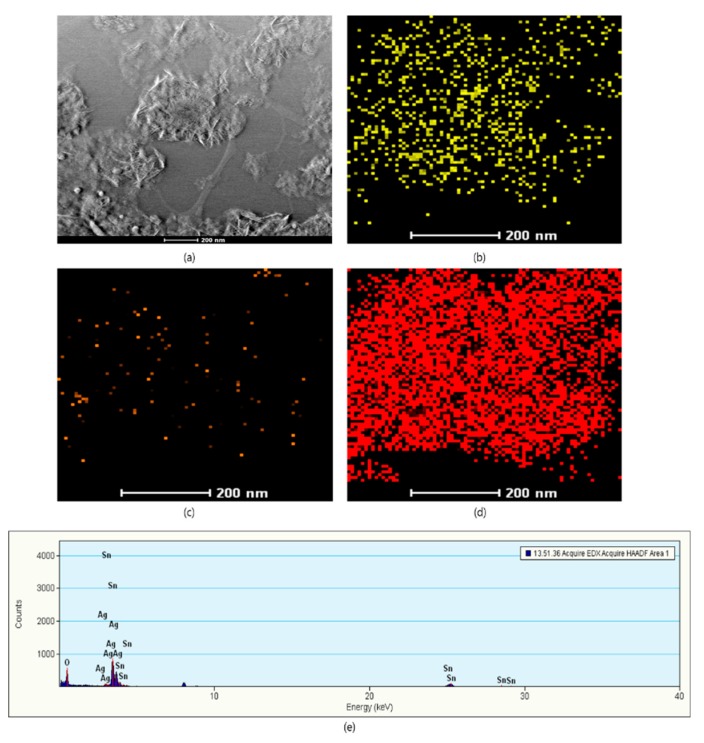
High-angle annular dark-field imaging (HAADF) and elemental mapping of Ag QDs/SnO_2_ nanostructures: (**a**) HAADF image; (**b**) Tin; (**c**) Silver;(**d**) Oxygen; (**e**) EDX spectrum of Ag QDs/SnO_2_ nanostructures.

The SEM images in Figure 2 illustrate the morphological appearances of pristine and Ag QD decorated SnO_2_ nanoflakes. Figure 2a,b depict the SEM images of SnO_2_ nanoflakes under different magnifications. Pristine SnO_2_ displays a flower architecture, consisting of large nanoflakes approximately 20 nm in thickness. Ag QDS/SnO_2_ nanostructures contain nanolamellae of Ag quantum dots, which were not observable by SEM (Figure 2c,d). Following Ag loading onto the SnO_2_ structure, there was no evidence of a morphological change in the SnO_2_ nanoflakes, which was further verified by high-magnification images (TEM images, Figure 3). Nonetheless, the presence of Ag in the composite was confirmed in the EDX spectrum represented in Figure 4. From the TEM images, it can be seen that the Ag QDs (<5 nm) were decorated among the SnO_2_ nanoflakes. Moreover, the Ag QDs were uniformly distributed throughout the SnO_2_ nanoflakes, suggesting the formation of heterostructures. In a typical high resolution TEM (HRTEM) image, lattice fringes of d = 0.26 nm spacing can be assigned to the Ag (111) planes, while the lattice fringes of d = 0.33 nm can be assigned to the (110) plane of SnO_2_. Furthermore, energy dispersive spectrometry (EDX) analysis suggested the existence of Ag, Sn, and O elements, as represented in Figure 5.

**Figure 6:**
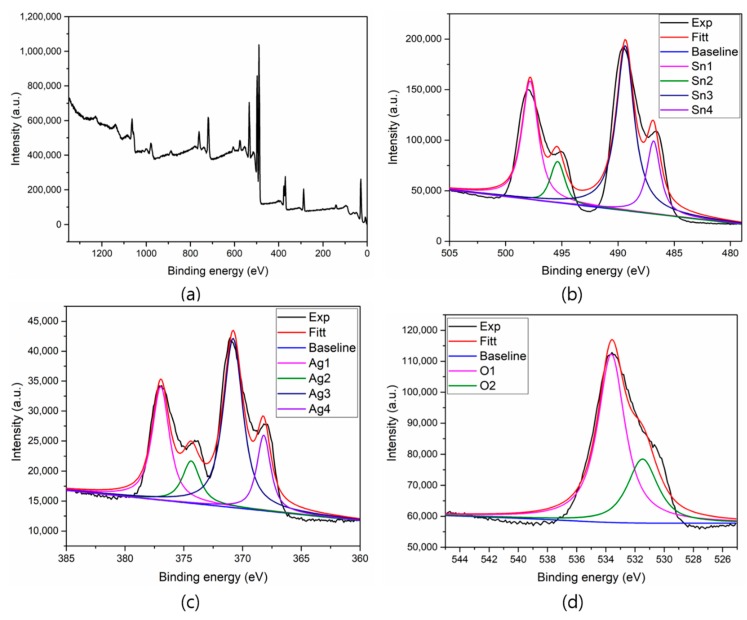
XPS spectra of the SnO_2_ and Ag QDs/SnO_2_ nanostructures (**a**) Survey scan, (**b**) Tin, (**c**) Silver, and (**d**) Oxygen elements.

The surface composition and oxidation states of Ag QD decorated SnO_2_ nanoflakes were analyzed through XPS (Figure 6). The presence of Sn 3d, Ag 3d, and O 1s was confirmed in the XPS survey scan. Here, we used a carbon contamination peak (284.8 eV) as an internal reference to estimate all remaining peaks. In Figure 6b, the spin orbit doublet peaks observed at approximately 497.95 and 489.61 eV were ascribed to the Sn 3d_3/2_ and Sn 3d_5/2_ binding energies (BEs), respectively. The BE difference between Sn 3d_3/2_ (497.95 eV) and Sn 3d_5/2_ (489.61 eV) was 8.34 eV, which clearly implies the presence of Sn^4+^ [24]. In addition, two satellite peaks appeared at 494. and 486.61 eV, which likely arose from the binding energy between Ag and Sn [16,24]. Furthermore, in Figure 6c, doublet peaks are visible at 376.95 and 371.09 eV, corresponding to the Ag 3d_3/2_ and Ag 3d_5/2_ BEs for Ag^0^ in the prepared sample. The small peaks at approximately 373.84 and 367.98 eV suggest Ag 3d BE in Ag QD decorated SnO_2_ nanoflakes [25]. The energy variation between Ag 3d_3/2_ and Ag 3d_5/2_ was 5.86 eV, indicating the establishment of metallic silver [26]. Small shifts in elemental BEs were observed for the Ag QD decorated SnO_2_ nanoflakes, which indicates that the solid interaction between Ag QDs and SnO_2_ nanoflakes arises from the transfer of an electron between Ag and SnO_2_. Figure 6d shows the O 1s spectra; the peak at 530.5 eV corresponds to the host lattice oxygen, and the peak at 533.5 eV implies the presence of hydroxyl groups (O_2_^−^, OH^−^) on the surface of the SnO_2_ nanoflakes [27,28,29].

**Figure 7:**
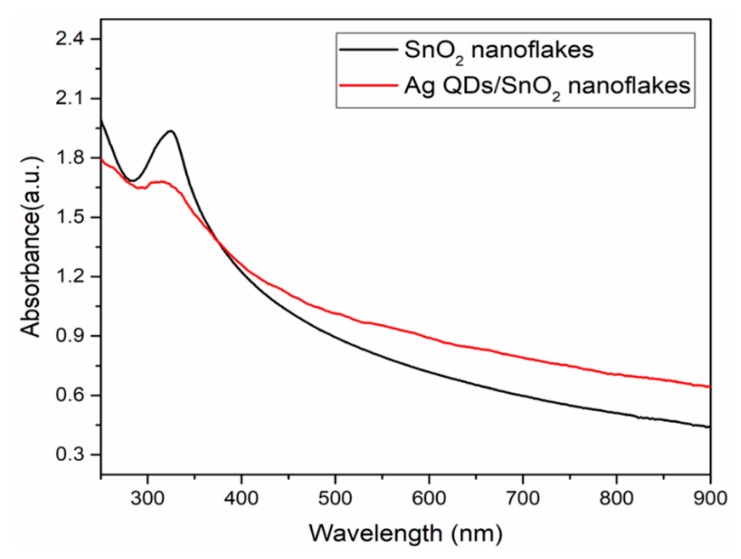
UV–Vis absorption spectra of the SnO_2_ and Ag QDS/SnO_2_ nanostructures.

The optical absorption properties of synthesized SnO_2_ and Ag QD decorated SnO_2_ nanoflakes are represented in Figure 7. SnO_2_ nanoflakes exhibited an absorption band in the UV region, while the Ag QD decorated SnO_2_ nanoflake absorption shifted toward a higher wavelength, and included tailing in the visible region. The SnO_2_ nanoflakes exhibited an absorption band at 320 nm in the UV region, while for the Ag QD decorated SnO_2_ nanoflakes, the absorption band shifted by 5 nm and the intensity of the band decreased. This can be attributed to the charge transfer between Ag and SnO_2_ nanoflakes, which is expected to bring about an enhancement in photocatalytic activity. Analysis of UV–Vis absorption spectra was performed in order to evaluate the bandgap of the prepared samples by using the simplified equation: α*hν* = A(*hν* − *E*g)^2^, where α is the absorption coefficient; ν is the frequency; A is the constant; and *E*g is the energy gap. The bandgap of SnO_2_ and Ag QD/SnO_2_ structures was 3.2 and 3.1 eV, respectively. The results suggest that the Ag–SnO_2_ nanoparticle size was smaller when compared to that of SnO_2_, due to the introduction of an impurity level developed in the bandgap of the composite, resulting in a reduction in electron transition energy. Moreover, absorption band shifts and optical bandgap reduction have an impact on the photo-induced electron-hole recombination rate, which influences photocatalytic activity. Furthermore, the SPR band of silver was attributed to the perpendicular vibrations of dipoles between particles and individual optical vibrations.

**Figure 8:**
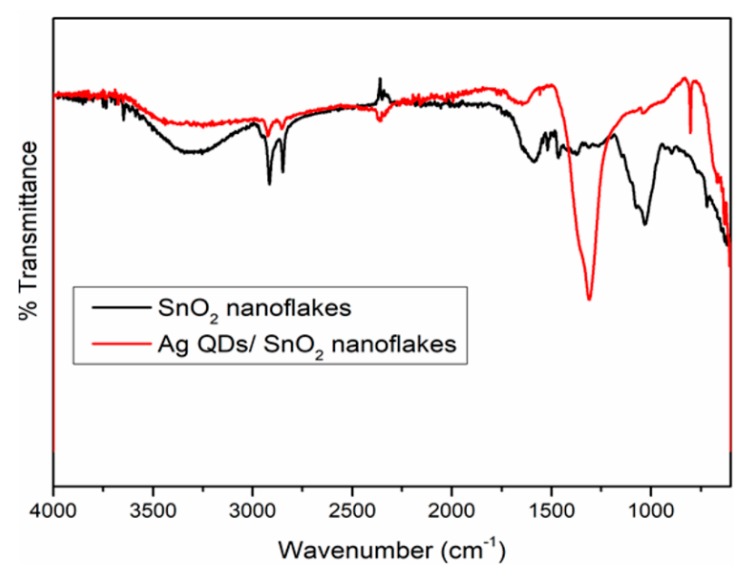
FTIR spectra of the SnO_2_ and Ag QDs/SnO_2_ nanostructures.

To examine the role of HDA in the preparation of SnO_2_ nanoflakes and Ag QD decorated SnO_2_ nanoflakes, FTIR spectra were recorded and analyzed, as illustrated in Figure 8. For both the SnO_2_ and Ag QDs/SnO_2_ samples, the peaks at 2918 and 2849 cm^−1^ represent the stretching vibrations of CH_3_, and symmetric and asymmetric C–H vibrations of CH_2_ in HDA, respectively. The peak at 1311 cm^−1^ in the Ag QDs/SnO_2_ sample represents the attachment of an N–H group onto the silver tin oxide nanostructures. Moreover, less intense peaks were observed in the region of 1200–1700 cm^−1^ in samples with HDA. The peaks shifted approximately 30 cm^−1^ due to surface interaction between the nanomaterials and HDA. The stretching vibrations in the region of 600–1000 cm^−1^ were attributed to the antisymmetric molecular vibrations of Sn–O–Sn and O–Sn–O [30].

**Figure 9:**
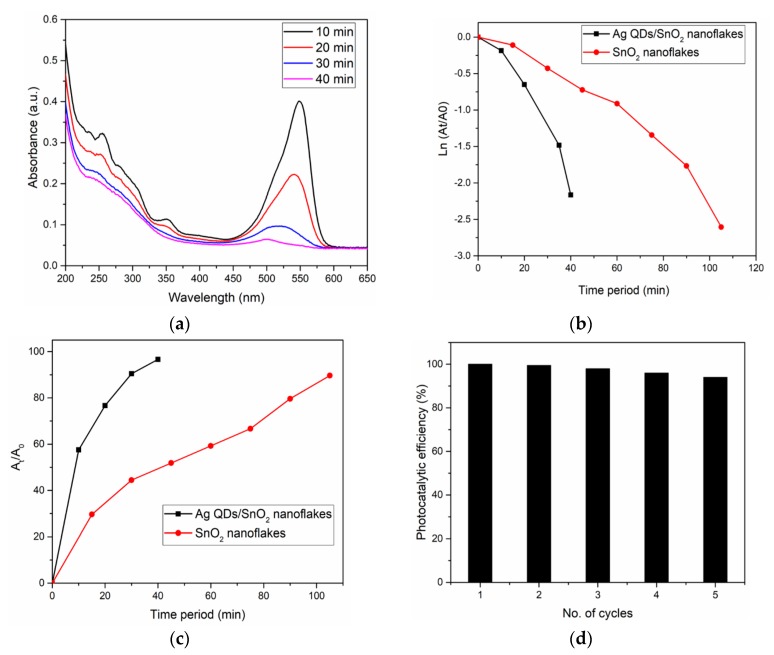
(**a**) Photocatalytic dye (RhB) degradation by the Ag QDs/SnO_2_ nanostructures. (**b**) Photocatalytic activity comparison of the Ag QDs/SnO_2_ and SnO_2_ nanostructures. (**c**) Reduction of RhB at specific time intervals with various catalysts. (**d**) Ag QDs/SnO_2_ nanoflake stability: photocatalyst recycling under light illumination.

The photocatalytic performances of the pristine and Ag QD decorated SnO_2_ nanoflakes were evaluated by studying RhB degradation under light irradiation. The kinetics of RhB degradation were determined by the pseudo-first order model. The results of the degradation by Ag QD decorated SnO_2_ nanoflakes are illustrated in Figure 9a. The rate of degradation was calculated using the following Langmuir–Hinshelwood pseudo-first order kinetic equation [31]

ln (*A*_t_/*A*_0_) = −k*t*

where k is the apparent rate constant, and *A*_0_ and *A*_t_ are the dye concentrations at the initial time and at time *t*, respectively. The photocatalytic reaction exhibits a linear relationship of photodegradation with respect to time (Figure 9b). From the results, it is evident that the reaction rate constant (k) is higher for the Ag QDs/SnO_2_ catalyst when compared to that for the SnO_2_ nanoflakes. Therefore, we can conclude that the degradation efficiency of the Ag QD decorated sample was higher than that of the pristine SnO_2_ nanoflakes.

Comparisons of the obtained results with recent literature [23,32,33,34,35,36,37,38,39,40] are shown in Table 1. In our case, under light illumination, RhB was 96% decomposed within 40 min (Figure 9c). This may be due to the plasmon effect of Ag QDs, which acts as an electron supplier to receive the photogenerated electrons. The stability toward the recycling of Ag QDs decorated SnO_2_ nanoflakes was studied for five cycles, and the results are shown in Figure 9d. Based on the recycling results, RhB decomposition was at 94% in the fifth cycle, which was marginally lower when compared to that in the first cycle. The decreasing efficiency of the photocatalyst can be attributed to the diminished surface interaction between the reaction intermediates in the dye oxidation process.

**Figure 10:**
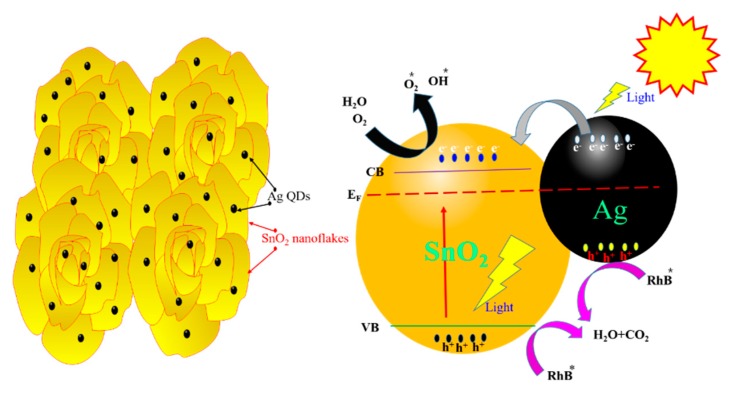
Schematic representation of the photocatalytic reaction progression and charge transfer of Ag QDs/SnO_2_ nanoflakes under light irradiation.

A plausible mechanism for the formation of photo-induced carriers and their separation, transportation, and associated reduction process of dye pollutants under light irradiation is shown in Figure 10. Under light irradiation, photonic energy was more than or equal to the bandgap of SnO_2_; the photo-induced generated electron-hole pair and work functions of SnO_2_ and silver differ, which creates a Schottky barrier between the metal and semiconductor in the nanocomposite [41]. Under light irradiation, Ag QDs/SnO_2_ was in the excited state because of the SPR of the Ag, and the generated electrons were transferred to the conduction band (CB) of SnO_2_. Dissolved oxygen molecules in water form superoxide radical anions (O_2_*), and hydroxyl radicals (OH*), which scavenge the electrons in the CB of SnO_2_. In the case of RhB degradation, the radical molecules react with the central carbon of RhB and initiate the deethylation process. Furthermore, primary oxides such as phthalic acid, adipic acid, and terephthalic acid are created form this intermediate state. The intermediate compounds degrade into smaller compounds such as propane-1,2,3-triol and butane-1,3 diols, among others. Ultimately, these compounds are mineralized to form H_2_O and CO_2_ [42].

## 4. Conclusions

In conclusion, we prepared silver quantum dot decorated 2D SnO_2_ nanoflakes for the photocatalytic abatement of water pollutants by using a straightforward and mild hydrothermal protocol. The Ag QD (<5 nm) decorated SnO_2_ and SnO_2_ nanoflakes exhibited a flower architecture, consisting of large 2D-nanoflakes approximately 20 nm in thickness. The photocatalytic activity of Ag QDs on the 2D-SnO_2_ nanostructures was determined to be double that of the 2D-SnO_2_ nanostructures. The association of Ag on SnO_2_ nanostructures may decrease contact resistance, which enhances light harvesting through plasmonic resonance by noble metals. Moreover, it suppresses the photo-induced electron-hole pair recombination rate, all of which lead to increased catalytic efficiency. Furthermore, we believe that this method of metal QDs-modified semiconductor synthesis can meet the requirements of industrial effluent treatment.

## Figures and Tables

**Table 1:** Comparison of the Ag QDs/ SnO_2_ nanoflakes photocatalytic activity with recent literature examples.

S. NO	Photocatalyst	Synthesis Method	Dye	Light Source	Degradation Time (min)	Ref.
1	Ag/SnO_2_ QDs	One-pot	RhB	Sunlight	180	[23]
2	SrO_2_/g-C_3_N_4_	Dry	RhB	Visible	60	[32]
3	Au/ SnO_2_ QDs	Solvothermal	RhB	Visible	200	[33]
4	α-Fe_2_O_3_/Ag/SiO_2_/SnO_2_	Template	RhB	UV & Visible	180	[34]
5	CdS QDs/TiO_2_	Electrochemical	RhB	Visible	300	[35]
6	g-C_3_N_4_/ SnO_2_	Refluxing	RhB	Visible	420	[36]
7	CeO_2_-QDs/Cu_2_O	Hydrothermal	RhB	Simulated sunlight	180	[37]
8	Fe_2_O_3_ Nanorods	Chemical	RhB	Simulated solar	270	[38]
9	SnO_2_	Chemical	RhB	UV light	270	[39]
10	Ag-TiO_2_-P25	Sonochemical	RhB	Simulated solar	100	[40]
11	Ag/ZnO	Chemical	RhB	Visible & UV	90	[41]
12	Ag QDs/SnO_2_	Hydrothermal	RhB	Simulated solar	40	(Present Study)
